# Contextual factors matter: A two-year exploration into the impact of contextual factors on elite women’s rugby sevens match-play movement demands

**DOI:** 10.1371/journal.pone.0322407

**Published:** 2025-05-07

**Authors:** Ross J. Brosnan, Denis Visentin, Greig Watson, Matthew Schmidt

**Affiliations:** 1 University of Tasmania, Hobart, Tasmania, Australia; 2 Murdoch University, Perth, Western Australia, Australia; Portugal Football School, Portuguese Football Federation, PORTUGAL

## Abstract

**Introduction and aims:**

Women’s rugby sevens is a rapidly growing sport that imposes unique match-play movement demands on participants. This research investigated the effect of contextual factors on the match-play movement demands of international and domestic women’s rugby sevens players.

**Methods:**

An observational, longitudinal study design was conducted to characterise Global Navigation Satellite Systems (GNSS) match-play movement demands in international (n = 23) and domestic (n = 42) players across two seasons and nine series of an elite domestic women’s dual-level rugby sevens tournament. In total, across the 65 players, 1461 matches were analysed. Match-play movement demands were assessed through distance, speed, and acceleration metrics using 10 and 15 Hz (5 Hz interpolated to 15 Hz) GNSS devices. Contextual factors were grouped into four themes: Player, Match, Tournament, and Environment. Data was analysed using univariate and multivariate mixed-effects regression.

**Results:**

Multi-variate regression identified that higher intensity match-play movement demands were associated with day 1, half 1, winning and/or drawing the match, closer score lines, playing in a top-5 ranked team, playing against opponents closer in the standings, starting the match, playing as a back or speed edge, being an international player, playing in warmer climates, and playing later in the day.

**Conclusion:**

This study highlights how contextual factors can affect match-play movement demands in women’s rugby sevens. These findings can help coaches tailor training, optimise tactical decisions, and manage player workloads more effectively.

## Introduction

Global navigation satellite system (GPS/GNSS) and accelerometer-based inertial sensors, henceforth referred to as GNSS, are commonly used to quantify match-play movement demands of team sports [1–4]. These types of demands include running, sprinting, changing direction, accelerating, and decelerating, all of which can be categorised by intensity [5,6]. Rugby sevens is one such sport, where research suggests that high intensity movement demands, reflecting high-intensity passages of play, can contribute to match outcomes such as winning [7–10]. Quantifying these demands and understanding how contextual factors influence them are essential for assessing athlete and team performance, as well as informing best practice for preparation and program delivery [7,11–13].

With the global rise of women’s rugby sevens [14], most research has focused on general physiological and match-play movement demands during match play, providing normative values [15,16]. However, these studies often overlook the various contextual factors—such as opponent rank, match conditions, or player roles—that can significantly influence these demands. This may result in one-dimensional conclusions that fail to account for the complexity of real-world competition, where match outcomes are influenced by multiple interacting variables [17–21]. Understanding these contextual factors can help to better evaluate match and tournament demands and performance, leading to more specific programs that replicate actual competition [13]. Although some studies have begun to specifically explore these factors in women’s rugby, they are limited in scope, often focusing on single teams [13], or specific isolated conditions [12,18,20,22,23]. This knowledge gap in literature is crucial to address and develop more effective training, preparation, and performance strategies tailored to the unique demands of women’s rugby sevens [15,16].

In men’s rugby sevens, individual and contextual factors—such as physical strength, position, opponent rank, scoreline, tournament day, game half, athlete rank, playing time, and game outcome—affect physiological profiles and total distance, relative distance, high-speed running distance, and accelerations [9,24–28]. Fewer studies have quantified the impact of isolated contextual factors on women’s rugby sevens match-play movement demands [12,13,18,20,22,23,29]. Normative research has shown differences in match-play movement demands between first and second half of play [12,22], international and domestic tournaments [23], and international and domestic players competing in the same tournament [29]. Goodale et al. [2017], which investigated a single international team across one tournament, is the only research article to date with a primary aim of investigating multiple contextual factors in women’s rugby sevens. This research found that game half (half 1 v half 2), game outcome (win v loss), tournament day (day 1 v day 2), opponent rank (top 4 world rank vs bottom 4 world rank), and margin of outcome (≥14 points v ≤14 points) affected match-play movement demands, with the game half also influencing physiological characteristics [13]. Comprehensive analysis of a larger range of contextual factors across multiple teams and seasons is therefore still lacking.

This study aimed to address this gap in the literature by determining the effect of contextual factors on the match-play movement demands of both international and domestic women’s rugby sevens players. By quantifying these demands in an elite dual-level tournament across multiple teams, positions, match types, and over two seasons, this study seeks to provide a more comprehensive analysis. The findings will help bridge the gap between international and domestic players and better inform talent identification, training prescription, load monitoring, and match preparation processes. This includes early identification of players related to positional preferences and characteristics, opportunities for re-loading injured players, managing loads of certain player types based on context, workloads, and intensities, and tactical insights as tournaments evolve.

## Materials and methods

### Experimental design

**Fig 1:**
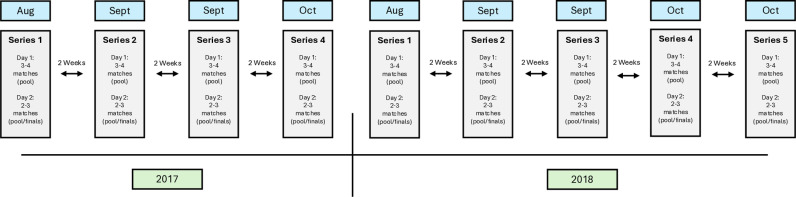
Timeline of the 2017 and 2018 dual-level tournaments.

A longitudinal observational study design was used to characterise and compare match-play movement demands in international and domestic women’s rugby sevens players. Data was collected from players in multiple teams during 9 series of an elite dual-level tournament in Australia, spanning 2017 (4 series) and 2018 (5 series). Each series consisted of 7 matches played over two days, with day one featuring 3–4 pool stage matches and day two consisting of 2–3 matches, including the final pool match and/or finals rounds. A two-week interval occurred between each series within the respective seasons (Fig 1). This tournament was used as a feeder tournament for international selection, with competing teams provided 2–4 contracted international players from the host nation to supplement squads of elite domestic talent. Players were categorised into three positional groups: backs, forwards, and speed edge players, as determined by domestic and national selection procedures. GNSS data was collected as part of the team’s routine sport science practice.

### Participants

All but two of the international players were part of the tournament host nation, a top 2 team during the 2017–2018 World Rugby Women’s Series standings and reigning Olympic Gold Medallists. The remaining international players were from top 12 teams in the 2017–2018 World Rugby Women’s Series standings. Domestic players who participated were deemed the top domestic talent from the host nation, many with ambitions for international honours, which made for a high-profile tournament. All players were provided with information regarding the study and provided written consent. The start of the recruitment period for the data collection was 1/07/17 and ran until 31/07/2018. Players and teams were recruited between 1/07/17 and 31/07/2018 with data collection occurring in the first year from 1/7/2017 to 31/07/2017 and in the second year 1/7/2018 to 31/07/2018. All data collection across the two seasons was completed by 31/10/2018. All procedures were approved by the Tasmanian Health and Medical Human Research Ethics Committee in line with the requirements stipulated in the Declaration of Helsinki; Ethics No: H0015313.

### Data collection procedures and contextual factors

Match-play movement demands were recorded using 27 commercially available GNSS units sampling at 10 Hz and 5 Hz and interpolated to 15 Hz (14 GPSports EVO unis & 13 GPSports HPU units, Catapult Innovations, Melbourne, Australia). Players were assigned the same device for each match when possible [30]. The validity, reliability, and agreement of these types of GNSS units have been confirmed [5,6,31,32]. GNSS units were activated 15 minutes before data collection to allow for satellite signal, and devices were placed in custom pouches between the scapulae within the player’s jersey. Data was downloaded and analysed by the respective GNSS brand consoles (HPU Software GPSports Firmware R1 2018.3; EVO Software GPSports – Version 1.5.9 Build #37195). Height and weight were also taken in routine sports science anthropometry screening at the beginning of the season.

For each game, the match-play movement demand variables were quantified relative to minutes of play for all players (Table 1). These types of variables and bands have been previously applied in studies covering multiple sports [1,2,6,7,12,18,22,23,30]. Contextual factors were logged for each match and player and were categorized into Tournament, the Match, the Player, and the Environment (Table 2).

**Table 1:** Match-play movement demands.

Match-Play Movement Demands	
**Distance Demands**	
Total Distance	(TD; m)
Low-Speed Distance	(LSD; 0–3.3 m·s^-1^)
Moderate-Speed Distance	(MSD; 3.3–6 m·s^-1^)
High-Speed Distance	(HSD; 6–7.5 m·s^-1^)
Very High-Speed Distance	(VHSD; > 7.5 m·s^-1^)
**Acceleration Demands**	
Moderate Intensity Acceleration Efforts	(MIAE; 2.0–2.5 m·s^-2^)
High Intensity Acceleration Efforts	(MIAE; > 2.5 m·s^-2^)
Moderate Intensity Deceleration Efforts	(MIDE; -2.0 - -2.5 m·s^-2^)
High Intensity Deceleration Efforts	(MIDE; > -2.5 m·s^-2^)
**Speed Demands**	
Maximum Velocity	(MV, m·s^-1^)

Abbreviations: m·s, meters per second.

**Table 2:** Contextual factors with the baseline reference level indicated.

Contextual Factors	
**The Tournament**	
Day Number	Day 1 (D1) Day 2 (D2) (Ref)
Halves of Play	Half 1 (H1) Half 2 (H2) (Ref)
Match Type	Pool (P) Final (F) (Ref)
**The Match**	
Result	Draw (D) Loss (L) Win (W) (Ref)
Score-line: Margin of Result	<14 Points (-14) >14 Points (+14) (Ref)
Own Team Ranking	Top 5 (T5) Bottom 5 (B5) (Ref)
Opponent Ranking Difference	Opponent >4 (1) Opponent >1–3 (2) Opponent <1–3 (3) Opponent <4 (4) (Ref)
**The Player**	
Playing Status	Starter (ST) Sub (SB) (Ref)
Player Position	Speed Edge (SE) Back (B) Forward (F) (Ref)
Player Level	Int (I) Dom (D) (Ref)
**The Environment**	
Temperature	Warm (W) Moderate (M) Cold (C) (Ref)
Game Time	Evening (E) Afternoon (A) Morning (M) (Ref)

Abbreviations: Ref, reference.

### Statistical analysis

GNSS data was exported using pre-built spreadsheets (Microsoft Excel 2016, Redmond, WA, USA) and only excluded for logistical errors such as device malfunctions. Ony data with adequate satellite signal strength (>6) were analysed. Separate univariate linear mixed-effect models with Bonferroni post-hoc tests initially assessed differences in match-play movement demands across contextual factors, addressing pseudo-replication within the dataset from within-player repeated measures. Specifically, linear mixed-effect models were conducted for each individual match-play movement demand to identify significant differences between the contextual factor levels. Each model included contextual factors as fixed effects and participant as random effects. This approach is suitable for data with dependent samples (e.g., multiple observations per subject) [33]. The analysis was undertaken using Statistical Package for Social Sciences (SBSS version 21, IBM, Armonk, NY), and data can be found in the supporting information (S1 File). Significant variables (P < 0.05) were analysed post hoc using a Tukey procedure to correct for type I errors for multiple comparisons. This information can be found in the appendices. Additionally, and reported within this paper, mixed-effects multivariate analyses were conducted as the contextual factors were not all independent, with correlations likely between some variables. Variables were removed using stepwise reduction from a full model with variables that were P ≥ 0.05 dropped at each step and repeated until all variables left in the model were P < 0.05. This stepwise reduction can also be found in the appendices in S6 Table.

## Results

A total of 65 women’s rugby sevens players contributed to the study with twenty-three international-level (5 speed edge, 10 backs, and 8 forwards; mean ± SD age 23.4 ± 3.5 y, height 168.6 ± 7.0 cm, mass 70.0 ± 5.4 kg), and forty-two domestic-level (12 speed edge, 13 backs, and 17 forwards; mean ± SD age 24.8 ± 4.0 y, height 168.8 ± 2.2 cm, mass 71.0 ± 9.5 kg). A total of 1461 player matches were included (domestic match-play data files = 860; min = 1; max = 84; mean = 22; international match-play data files = 601; min = 3; max = 87; mean = 26). Mean ± SD for all relative match play movement demands per minute are presented in the supporting information, S1 Table, the univariate analysis for each set of contextual factors is presented in S2–S5 Tables, and the multivariate analysis results are presented below and in Table 3.

**Table 3:** The Tournament, The Match, The Player, The Environment (Mean >, Mean % Dif, Standard Deviation, 95% Upper Lower CI, P-Value).

		Multivariate Analysis									
		Distance					Acceleration				Speed
	%MD, (SE), [95% CI L, U], P	Total Distance	Low Speed Distance	Moderate Speed Distance	High Speed Distance	Very High-Speed Distance	Moderate Intensity Acceleration Efforts	High Intensity Acceleration Efforts	Moderate Intensity Deceleration Efforts	High Intensity Deceleration Efforts	Maximal Velocity
The Tournament	Day Number Day 1 (D1) Day 2 (D2) (Ref)	D1 > D2, 3.03 (2.02), [2.45, 5.23], P < 0.001		D1 > D2, 7.66 (2.28), [1.56, 3.81], P < 0.001	D1 > D3, 12.11 (0.54), [0.02, 1.05], P = 0.042						
	Halves of Play Half 1 (H1) Half 2 (H2) (Ref)							H1 > H2, 15.64 (0.03), [0.007, 0.05], P = 0.006		H1 > H2, 8.09 (0.04), [0.00, 0.07], P = 0.035	H1 > H2, 4.04 (0.20), [0.16, 0.32], P < 0.001
	Match Type Pool (P) Final (F) (Ref)										
The Match	Result: Draw (D) Loss (L) Win (W) (Ref)	L > W, 2.34 (1.57), [-3.55, -0.24], P = 0.025	L > W, 1.83 (0.77), [-1.99, -0.38], P = 0.004								
	Score-line: Margin of Result <14 Points (-14) >14 Points (+14) (Ref)					-14 > 14 + , 41.62 (0.24), [0.11, 0.51], P = 0.003		-14 > 14 + , 18.03 (0.03), [0.002, 0.52], P = 0.030		-14 > 14 + , 10.19 (0.04), [0.004, 0.07], P = 0.028	-14 > 14 + , 3.88 (0.19), [0.09, 0.27], P < 0.001
	Own Team Ranking Top 5 (T5) Bottom 5 (B5) (Ref)	T5 > B5, 2.68 (1.80), [0.57, 4.12], P = 0.010				T5 > B5, 63.39 (0.41), [0.26, 0.695], P < 0.001	T5 > B5, 19.31 (0.06), [0.04, 0.11], P < 0.001	T5 > B5, 44.99 (0.08), [0.02, 0.81], P < 0.001		T5 > B5, 21.46 (0.10), [0.04, 0.12], P < 0.001	T5 > B5, 9.09 (0.45), [0.35, 0.57], P < 0.001
	Opponent Ranking Difference Opponent >4 (1) Opponent >1–3 (2) Opponent <1–3 (3) Opponent <4 (4) (Ref)			2 > 4, 3.55 (0.73), [0.46, 3.56], P = 0.011							
		3 > 4, 3.73 (2.47), [1.87, 5.66], P < 0.001		3 > 4, 7.76 (1.63), [0.1.63, 4.52], P < 0.001							
The Player	Playing Status Starter (ST) Sub (SB) (Ref)		ST > SB, 2.11 (0.88), [0.21, U: 2.23], P = 0.018						SB > ST, 10.09 (0.03), [-0.07, -0.01], P = 0.025		
	Player Position AU I.D. Speed Edge (SE) Back (B) Forward (F) (Ref)		SE > F, 2.25 (0.96), [0.32, 2.36], P = 0.010	F > SE, 20.77 (4.16), [-7.11, -4.23], P < 0.001	SE > F, 52.48 (1.71), [1.61, 2.897], P < 0.001	SE > F, 144.85 (1.08), [1.24, 1.74], P < 0.001	F > SE, 13.37 (0.04), [0.01, 0.09], P = 0.007	SE > F, 29.05 (0.05), [0.01, 0.68], P = 0.022		SE > F, 23.71 (0.10), [0.08, 0.16], P < 0.001	SE > F, 11.31 (0.55), [0.57, 0.82], P < 0.001
			F > B, 2.37 (0.99), [-2.59, -0.75], P < 0.001		B > F, 32.96 (0.95), [0.63, 1.78], P < 0.001	B > F: 86.05 (0.31), [0.06, 0.51], P = 0.015	B > F, 20.02 (0.06), [0.04, 0.10], P < 0.001	B > F, 29.77 (0.05), [0.003, 0.06], P = 0.031		B > F, 26.46 (0.11), [0.09, 0.17], P < 0.001	B > F, 6.74 (0.32), [0.18, 0.40], P < 0.001
	Player Level Int (I) Dom (D) (Ref)			I > D, 6.58 (1.38), [-2.71, -0.35], P = 0.011	I > D, 24.71, (0.80), [0.28, 1.32], P = 0.002			I > D, 59.54 (0.11), [0.96, 0.15], P < 0.001		I > D, 18.64 (0.08), [0.02, 0.095], P = 0.002	I > D, 7.28 (0.36), [0.10, 0.31], P < 0.001
The Environment	Temperature Warm (W) Moderate (M) Cold (C) (Ref)		C > W, 2.94 (1.22), [-2.26, -0.06], P = 0.039			W > C, 43.61 (0.25), [0.12, 0.65], P = 0.005		W > C, 14.17 (0.02), [0.007, 0.72], P = 0.018			
						M > C, 36.44 (0.09), [0.00, 0.44], P = 0.048		M > C, 14.17 (0.01), [0.01, 0.06], P = 0.015			
	Game Time Evening (E) Afternoon (A) Morning (M) (Ref)							E > M, 21.21 (0.04), [-0.03, 0.02], P = 0.007		E > M, 9.58 (0.04), [0.00, 0.093], P = 0.040	

*The bolding shows the direction of the effect. The ‘>’ or ‘<’ signs and green shading show significance. Grey shading also shows significant univariate variables that were removed throughout the multivariate reductionist process.

### The Tournament: (Day Number, Halves of Play, Match Type)

For the contextual factor **day number**, total distance (%MD, 3.03, P < 0.001) moderate-speed distance (%MD, 7.55, P < 0.001), and high-speed distance (%MD, 12.11, P = 0.042) were all significantly higher on day 1 compared to day 2, particularly high-speed distance. In terms of **halves of play**, high-intensity acceleration efforts (%MD, 15.64, P = 0.006), high-intensity deceleration efforts (%MD, 8.09, P = 0.035), and maximal velocity (%MD, 4.04, P < 0.001) were also significantly higher in Half 1, particularly high-intensity acceleration efforts. **Match type** had no significant effect on any variables.

### The Match: (Own Team Ranking, Score-Line, Match Outcome, Ranking Difference)

With respect to **match outcomes** (Total Distance; %MD 2.34, P = 0.042, low speed distance; %MD 12.11, P = 0.042) and **ranking difference**, (moderate speed distance; %MD 12.11, P = 0.042), there were significant but trivial effects on low intensity match-play movement demands and no effect on high intensity variables. **Score-line** (very high speed distance; %MD 41.62, P = 0.003, high-intensity acceleration efforts %MD 18.03, P = 0.030, high-intensity deceleration efforts; %MD 10.19, P = 0.028, maximal velocity; %MD 3.88, P < 0.001), and **ranking difference** (very high speed distance; %MD 63.39, P < 0.001, high-intensity acceleration efforts %MD 19.31, P < 0.001, high-intensity deceleration efforts; %MD 21.46, P < 0.001, maximal velocity; %MD 9.09, P < 0.001), significantly influenced match-play movement demands, with high intensity movement variables expressed more in top ranked teams and in games with scoring margins less than 14 points.

### The Player: (Playing Status, Player Position, Player Level)

The contextual factors characterising the players had significant effects on high intensity game movement demands related to distance and acceleration. With respect to **player level**, international players expressed greater high intensity acceleration (%MD 59.54, P < 0.001) and deceleration efforts (%MD 18.64, P = 0.002) along with a higher maximum velocity (%MD 7.28, P < 0.001). **Player position** also significantly affected match-play movement demands, with speed edges and backs displaying greater high intensity movement demands than Forwards (Table 3). Regarding **playing status**, substitutes did not significantly differ in movement demand variables than starters.

### The Environment: (Temperature, Game-Time)

**Temperature** influenced high-speed performance, with both warmer and moderate temperatures showing significantly higher very high-speed distance (%MD 43.61, P = 0.005), and high-intensity acceleration efforts (%MD 14.17, P = 0.015) than colder temperatures. With respect to **game-time**, high-intensity acceleration efforts (%MD 21.21, P = 0.007) and high-intensity deceleration efforts (%MD 9.58, P = 0.040) were also significantly higher in evening games when compared to morning games.

## Discussion

This study provides new insights into the impact of contextual factors on match-play movement demands among international and domestic women’s rugby sevens players. By analysing data across multiple teams, positions, players, and match types in a two-year elite dual-level tournament, we offer a comprehensive perspective on how tournament, match, player, and environmental factors influence performance. These findings are important for optimising preparation, practice, and strategic planning in women’s rugby sevens.

Tournament day number and half of play both impacted high intensity movement demands with reduced performance observed on day two of the tournament and in the second half of matches. With respect to day number, these results differ from previous research on international women’s [13] and men’s [34] rugby sevens players where movement demands were maintained throughout. The contrasting results may be due to players in previous research all being at international level and therefore exhibiting superior anaerobic and aerobic endurance capacity [35]. At international level playing ability, the quality and quantity of between-match recovery strategies employed, such as active/passive recovery, cryotherapy/cold-water immersion, compression, self-myofascial release, nutritional and hydration interventions and sleep, may also be more resourced and effective in sustaining performance over a multi-day tournament compared to elite domestic players [7].

The reduction in high intensity movement demands observed in the second half is consistent with previous research on international women’s [13,23] and men’s [28,36–38] rugby sevens, rugby league [39] and soccer [40]. This may be caused by residual central [41] or peripheral [42] fatigue during games and may be exacerbated in rugby sevens by the two-minute halftime interval preventing complete recovery and maintaining the same intensity from first to second half. Another contribution to the observed reduction in high intensity actions from half 1 to half 2 may also be due to the margin of the game ceasing to be competitive with teams consciously decreasing their levels of effort if they know with near certainty the outcome of the game.

Match outcome and opponent ranking had mixed effects on match play movement demands with more low intensity movement demands prevalent, compared to score margin and team ranking, which reflected differences in higher intensity movement demands. The effect of match outcome is consistent with previous men’s and women’s rugby sevens [24,43] and women’s rugby sevens [13] research for TD and LSD, in addition to rugby league [44,45], AFL [46], and soccer [47]. In contrast, own team ranking and a smaller scoring margin increased the expression of high intensity match-play movement demands. This result with respect to team ranking is consistent with previous findings where actions which determine outcomes of matches have been linked directly with maximum effort running and high intensity passages of play [7–10]. Previous reports on the effect of scoring margin also found that high speed running demands increased when score lines were close in male rugby sevens [24], while total distance and moderate- and high-speed running distance were trivially greater when a team lost by a margin of 2 converted tries (14 points) in female rugby sevens, compared with a team who won by a similar margin [13].

Integrating these findings suggest that players playing in teams that are winning may possess efficient technical and tactical execution, and therefore needing lower running distances, while higher intensity differences, similar to own team rankings, may be due to players on winning teams having the ability to finish actions which determine outcomes of matches [7–10]. These results suggest that high-intensity efforts are crucial during high-stakes scenarios, especially when games are closely contested. Coaches can use these insights to develop game strategies that emphasise sustaining high-intensity efforts during critical phases of play, through methods such as simulation training strategies, and planned scenarios manipulated by different types of constraints, particularly in preparation for top-ranked teams or for predicted closely matched contests.

The use of substitute players appears anecdotally efficacious as a tactical means of maintaining team work-rate [34], with the replacement of one or more players at key stages of a match potentially influencing the match result, in a tactical strategy known as the ‘super sub’. However, moderate intensity match-play movement demands expressed by substitutes in this study were lower than starters, while there was no difference in higher intensity match-play movement demands. This is surprising as previous research in men’s rugby sevens contrasts these results and found that substitutes who came on in the 2nd half expressed higher intensity match play movement demands than starters [24]. The decrement in substitute performance we observed may result from the substitutes finding it difficult to acclimatise to the speed of the game, or not able to produce higher match play movement demands – possibly due to their ability, or preparation. The fact that substitutes did not differ significantly from starters in higher intensity movement demands suggests that training should also focus on preparing substitutes to perform effectively when introduced into high-intensity phases of play, to maintain team intensity.

Comparison of match-play movement demands between player position revealed that forwards showed significantly higher numbers in lower and moderate intensity movement demands when compared to backs and speed edge players. However, backs and speed edges showed several significantly higher numbers in higher intensity movement demands when compared to forwards, emphasising the position-specific nature of rugby sevens. Previous research into positional characteristics of women’s rugby sevens also demonstrated contrasting results at both international level and dual-tournament level with some research showing significant differences between backs and forwards, and other research showing no differences [12,13,48]. Only one research study to date has categorized players by three positional groups of backs, forwards, and speed edge players, determined by domestic and international selection procedures [29]. This research revealed significant differences between all positions and is consistent with the current study’s findings. These findings underscore the need for position-specific training regimens that cater to the distinct demands of Speed Edges, Backs, and Forwards. Speed Edges, who exhibited the greatest high-intensity demands, may benefit from targeted training to maximise their ability to sustain repeated high-intensity efforts, while the introduction of monitoring impacts and heart rate may be an important variable for forwards to gauge their workload and intensity, rather than focusing solely on movement.

International players also increased high intensity match-play movement demands compared to domestic level players in the same environment, similar to previous match-play data comparing tournament level [18,22,23]. This may reflect both the athlete’s physical capabilities but also the tactical match-play knowledge, strategy and anticipation of the play, which may be more advanced in international players. This may mean they are able to place themselves in positions where they can express higher intensity variables or attain velocities that domestic players are unable to [27]. International level players may also break the first line of defence more frequently which would create the opportunity to express higher velocity zones in open field running – something future research should determine.

To date there is limited research investigating the effects of temperature on elite women’s rugby sevens. There is reason to believe temperature may affect performance given that despite the very short game duration, players experience high peak core temperature values approaching thresholds that are associated with exertional heat illnesses and exceeding thresholds associated with hindered repeated sprint performance [49,50]. We observed that warm and moderate game time temperature increased some high intensity movement relative to cold temperatures. This is an unexpected finding if core temperature limits performance. This differs from previous work in soccer and football that suggests that decreases in match-play movement demands, particularly higher intensity, may be consciously or sub-consciously employed by players to allow maintenance of skill succession rate, with players modifying movement patterns to preserve technical actions [51–54]. To our knowledge this is also the first research paper looking at the time of day of matches in women’s rugby sevens. We observed a significant effect of game time with higher high-intensity acceleration and deceleration loads occurring later in the day compared to earlier.

Our results highlight the need for tailored preparation strategies based on environmental conditions. Coaches should account for the increased intensity demands in warmer and evening conditions and adjust physical preparation, hydration, and recovery protocols accordingly. Pre-match priming and post activation potentiation strategies, which have been shown to be common practice in preparation for optimal performance in team sports [7], could be adapted to help players acclimate to these environmental factors and perform optimally under varied conditions. Hydration and climate management strategies to account for warmer or colder temperatures could also help advance strategies that coaches can investigate.

Match-play movement demands in women’s rugby sevens are influenced by a multitude of factors including the dynamic demands of the individual games, the capacity of players on a given day to regulate their performance, and the unknown effects of team tactics and officiating style [34]. Due to this variability, a strength of the current study is the large data set comprised of both international and domestic women’s rugby sevens players, multiple teams, seasons, positions, and matches. This study design has been a consistent recommendation and throughout previous similar research [15,16,43,55,56]. Certain limitations of this study remained including weather and game-time only being assessed on a three-point scale. Additionally, the inclusion of data on skill notational analysis, and player and coach perceptions, cognitive demands such as motivation, anxiety, mental effort, and fatigue to coincide with match-play movement demands data will give a more detailed and holistic understanding of the influence of contextual factors on match-play performance.

Practical applications from this research include re-loading injured players from return to play protocols or managing loads of certain player types on days of matches. Tactical insights on how to get the ball to speed edge players with more space may also be implemented and realized as tournaments carry into day two [57]. Our study did not assess recovery methods, but the observed decline in performance highlights the importance of robust recovery and conditioning protocols, especially as fatigue from consecutive matches affects movement demands [7,15,16]. Future research should focus on recovery strategies that mitigate performance and intensity drops, particularly in multi-day tournaments and late-stage matches. Physical preparation and priming strategies may also be explored for adequate preparation for early morning game times, and for substitutes entering high intensity periods of matches [7]. Future analyses on the impact of temperature on match-play movement demands would benefit from integrating technical data, player hydration status, and core temperature, where possible, to identify if changes in match-running are to preserve technical ability.

Results may also be important for talent identification related to positional preferences and characteristics. Decisions on positional changes at an earlier time point in player development pathways may be an alternative option if existing players are unable to reach higher intensity demands of what is required at international or dual-level tournament characteristics in their current position. A focus on high intensity match-play movement demands—such as speed, acceleration, deceleration, and high intensity running—can be objective performance markers that could help in their pursuit of higher honours, and the identification of players that have existing attributes, or potential to express high intensity match-play movement demands may impact talent identification procedures.

Other practical applications include designing training preparation of high intensity demands for players to be able to acclimatise and supersede in competition [57]. A more holistic approach to match-play and tournament monitoring should also be considered which collates player data surrounding cognitive and perceptual demands such as motivation, anxiety, mental effort, and fatigue, and how they interact with match-play movement demands throughout tournaments [58–61]. Further research into cognitive and perceptual demands alongside physical performance could further refine training and match preparation strategies, offering a more complete view of player demands in women’s rugby sevens.

This study demonstrated the significant effect of contextual factors on game movement demands using data from multiple teams, seasons and players. This large data set reduces the biases inherent in studies with smaller data sets. These results advance our understanding of these contextual factors and provide practical insights for improving training, periodization, and player preparation. Coaches and practitioners can use these findings to better manage performance across various contexts, from domestic to international competitions.

### Practical applications

- Coaches and performance staff can prepare for faster games played in warmer weather conditions, played later in the day, during first halves, on day 1 of tournaments, played with or against more international level players, for backs and speed edge positions, when starting a game, when playing in a top 5 team, when playing against teams close in the standings, and when playing in a game with a close result.
- Coaches and performance staff have a framework of the match-play movement demands of players in higher ranked teams, international level athletes, and for specific positions. This can inform talent identification processes and help guide player development pathways and improve positional decisions.
- Coaches and performance staff can also use this information to aid in the preparation and simulation of scenarios in training which may replicate higher match-play movement demands in actual competition. This may help players acclimate to similar scenarios they may face during competition.
- As substitutes did not show significant differences in movement demands compared to starters, integrating substitutes effectively into high-intensity periods of play is essential for future research. Coaches and performance staff should manage and plan for substitutes to match the intensity of ongoing play and contribute effectively to the team’s performance and intensity.
- Coaches and performance staff members have can also use this information in assisting the monitoring of player loads, individual recovery, and developing more holistic return to play protocols.

## Supporting information

S1 Table Descriptive Stats. (DOCX)

S2 Table Uni-V - The Tournament. (DOCX)

S3 Table Uni-V - The Match. (DOCX)

S4 Table Uni-V - The Player. (DOCX)

S5 Table Uni-V - The Environment. (DOCX)

S6 Table post Review - Multi V Reductions. (DOCX)

S1 File Contextual Factors Data. (SAV)
