# Supplementary material for: Contextual factors matter: A two-year exploration into the impact of contextual factors on elite women’s rugby sevens match-play movement demands
Source: PLoS One. 2025 May 7;20(5):e0322407. doi: 10.1371/journal.pone.0322407 (PMC12057925; doi:10.1371/journal.pone.0322407)
Supplement: S1 Table — (DOCX) [file pone.0322407.s001.docx]

Table 1. Descriptive statistics of contextual factors (‘The Tournament’ and ‘The Match’) and outcome variables used in the multivariate analysis (Mean, Standard Deviation, 95% Upper, Lower CI).

|  |  |  | | | | | | | | | |
| --- | --- | --- | --- | --- | --- | --- | --- | --- | --- | --- | --- |
|  |  | **Distance** | | | | | **Acceleration** | | | | **Speed** |
|  | *M, (SE), [95% CI L, U]* | **Total Distance**  **(m)** | **Low Speed Distance**  **(m)** | **Moderate Speed Distance**  **(m)** | **High Speed Distance**  **(m)** | **Very High-Speed Distance**  **(m)** | **Moderate Intensity Acceleration Efforts**  **(#)** | **High Intensity Acceleration Efforts**  **(#)** | **Moderate Intensity Deceleration Efforts**  **(#)** | **High Intensity Deceleration Efforts**  **(#)** | **Maximal Velocity**  (m·s) |
| **The Tournament** | **Day Number**  Day 1    Day 2 | 95.77 (13.59),  [94.85, 96.70]  92.92 (12.29),  [91.92, 93.92] | 59.43 (7.78),  [58.87, 59.99]  59.44 (7.61),  [58.83, 60.05] | 30.88 (11.20),  [30.09, 31.67]  28.61 (9.81),  [27.75, 29.46] | 4.70 (5.23),  [4.36, 5.05]  4.17 (4.47),  [3.79, 4.55] | 0.82 (1.90),  [0.67, 0.96]  0.81 (1.98),  [0.65, 0.97] | 0.43 (0.29),  [0.41, 0.45]  0.44 (0.27),  [0.42, 0.46] | 0.25 (0.26),  [0.24, 0.27]  0.23 (0.22),  [0.21, 0.25] | 0.37 (0.29),  [0.35, 0.39]  0.38 (0.27),  [0.36, 0.40] | 0.63 (0.36),  [0.60, 0.65]  0.61 (0.32),  [0.59, 0.64] | 6.92 (0.95),  [6.85, 6.99]  6.85 (0.96),  [6.76, 6.93] |
|  | **Halves of Play**  Half 1  Half 2 | 95.20 (11.97),  [94.25, 96.15]  93.69 (14.01),  [92.76, 94.61] | 59.75 (7.30),  [59.19, 60.31]  59.10 (8.05),  [58.55, 59.65] | 30.10 (9.52),  [29.32, 30.87]  29.57 (11.60), [28.80, 30.33] | 4.57 (4.05),  [4.22, 4.92]  4.33 (5.59),  [3.99, 4.67] | 0.92 (1.97),  [0.77, 1.06]  0.72 (1.90),  [0.59, 0.86] | 0.44 (0.25),  [0.42 0.46]  0.43 (0.31),  [0.41, 0.45] | 0.26 (0.22),  [0.25, 0.28]  0.22 (0.26),  [0.21, 0.24] | 0.38 (0.23),  [0.36, 0.40]  0.37 (0.32), [0.35,0.39] | 0.64 (0.30),  [0.62, 0.67]  0.59 (0.38),  [0.57, 0.62] | 7.03 (0.91),  [6.96, 7.10]  6.75 (0.97),  [6.68, 6.82] |
|  | **Match Type**  Pool  Final | 95.36 (13.5),  [94.49, 96.22]  93.99 (12.24),  [91.88, 94.11] | 59.58 (7.88),  [59.05, 60.10]  59.19 (7.39),  [58.51, 59.86] | 30.50 (11.04), [29.76, 31.24]  28.76 (9.86),  [27.80, 29.71] | 4.55 (5,09),  [4.23, 4.88]  4.30 (4.57),  [3.88, 4.72] | 0.80 (1.93),  [0.66, 0.94]  0.83 (1.93),  [0.66, 1.01] | 0.43 (0.29),  [0.41, 0.45]  0.44 (0.27),  [0.42, 0.47] | 0.24 (0.26),  [0.23, 0.26]  0.25 (0.23),  [0.22, 0.27] | 0.38 (0.30),  [0.36, 0.39]  0.37 (0.25),  [0.35, 0.39] | 0.61 (0.36),  [0.59, 0.64]  0.63 (0.32),  [0.60, 0.66] | 6.88 (0.95),  [6.81, 6.95]  6.90, (0.96),  [6.81, 6.99] |
| **The Match** | **Result:**  Loss  Draw  Win | 93.44 (12.60), [92.49, 94.38]  95.36 (12.60), [91.81, 98.90]  95.65 (13.41), [94.62, 96.69] | 58.98 (7.61),  [58.41, 59.54]  58.37 (9.30),  [56.24, 60.51]  60.07 (7.62),  [59.45, 60.69] | 29.67 (9.39),  [28.86, 30.47]  30.54 (9.84),  [27.49, 33.59]  30.02 (11.64), [29.14, 30.90] | 4.13 (5.50),  [3.78, 4.48]  5.13 (4.40),  [3.81, 6.46]  4.80 (4.35),  [4.41, 5.18] | 0.73 (1.93),  [0.58, 0.88]  1.24 (2.08),  [0.68, 1.80]  0.88 (1.92),  [0.72, 1.04] | 0.43 (0.30),  [0.41, 0.45]  0.43 (0.26),  [0.35, 0.50]  0.45 (0.27),  [0.43, 0.47] | 0.21 (0.27),  [0.20, 0.23]  0.31 (0.26),  [0.24, 0.38]  0.28 (0.22),  [0.26, 0.30] | 0.37 (0.30),  [0.35, 0.39]  0.38 (0.26),  [0.31, 0.45]  0.38 (0.27),  [0.36, 0.40] | 0.60 (0.35),  [0.57, 0.62]  0.68 (0.47),  [0.59, 0.77]  0.64 (0.32),  [0.62, 0.67] | 6.79 (0.94),  [6.72, 6.87]  7.09 (0.95),  [6.80, 7.37]  6.98 (0.96),  [6.90, 7.06] |
|  | **Score-line: Margin of Result**  <14 Points  >14 Points | 94.97 (12.53), [94.00, 95.95]  93.99 (13.59), [93.02, 94.95] | 59.67 (7.72),  [59.09, 60.26]  59.20 (7.68),  [58.62, 59.78] | 29.54 (10.20), [28.70, 30.37]  30.16 (11.06), [29.34, 30.98] | 4.84 (4.56),  [4.48, 5.21]  4.08 (5.19),  [3.72, 4.44] | 0.98 (2.09),  [0.83, 1.14]  0.65 (1.76),  [0.50, 0.80] | 0.44 (0.28),  [0.42, 0.46]  0.43 (0.29),  [0.41, 0.45] | 0.27 (0.24),  [0.25, 0.29]  0.22 (0.25),  [0.20, 0.24] | 0.37 (0.25),  [0.35, 0.39]  0.38 (0.30),  [0.36, 0.40] | 0.65 (0.33),  [0.63, 0.68]  0.59 (0.35),  [0.56, 0.61] | 7.02 (0 .94),  [6.94, 7.10]  6.75 (0 .95),  [6.68, 6.83] |
|  | **Own Team Ranking**  Top 5  Bottom 5 | 96.20 (11.84), [95.00, 97.41]  93.66 (13.54), [92.83, 94.49] | 59.63 (7.39),  [58.89, 60.36]  59.34 (7.85),  [58.84, 59.84] | 30.27 (8.65),  [29.23, 31.31]  29.65 (11.46), [28.94, 30.37] | 5.14 (4.31),  [4.69, 5.60]  4.14 (5.13),  [3.83, 4.45] | 1.21 (2.13),  [1.02, 1.40]  0.63 (1.81),  [0.50, 0.76] | 0.49 (0.30),  [0.47, 0.52]  0.41 (0.27),  [0.39, 0.43] | 0.32 (0.28),  [0.30, 0.35]  0.21 (0.22),  [0.19, 0.22] | 0.39 (0 .31),  [0.36, 0.41]  0.37 (0.26),  [0.35, 0.39] | 0.71 (0 .33),  [0.68, 0.74]  0.57 (0.83),  [0.55, 0.60] | 7.31 (0.83),  [7.22, 7.41]  6.68 (0.95),  [6.62, 6.74] |
|  | **Opponent Ranking Difference**  Opponent >4  Opponent >1-3  Opponent <1-3  Opponent <4 | 95.00 (13.22), [92.63, 97.35]  95.11 (12.64), [93.86, 96.36]  95.42 (13.27), [94.32, 96.53]  91.93 (12.96), [90.53, 93.33] | 59.07 (7.77),  [57.65, 60.49]  60.09 (7.60),  [59.33, 60.84]  59.31 (7.78),  [58.65, 59.98]  58.93 (7.63),  [58.08, 59.77] | 30.20 (8.41),  [28.19, 32.20]  29.56 (9.98),  [28.49, 30.62]  30.83 (11.10), [29.89, 31.77]  28.53 (11.28), [27.33, 29.72] | 4.79 (5.19),  [3.91, 5.67]  4.61 (4.39),  [4.14, 5.08]  4.66 (5.58),  [4.25, 5.07]  3.82 (),4.12  [3.30, 4.34] | 0.97 (1.87),  [0.60, 1.34]  0.93 (2.03),  [0.74, 1.13]  0.76 (1.85),  [0.59, 0.93]  0.69 (1.96),  [0.47, 0.91] | 0.48 (0.34),  [0.43, 0.53]  0.45 (0.30),  [0.43, 0.48]  0.42 (0.27),  [0.40, 0.45]  0.41 (0 .25),  [0.39, 0.44] | 0.32 (0.36),  [0.28, 0.37]  0.26 (0.24),  [0.24, 0.20]  0.24 (0.23),  [0.22, 0.26]  0.20 (0.21),  [0.17, 0.22] | 0.40 (0.43),  [0.36, 0.45]  0.38 (0.26),  [0.36, 0.40]  0.37 (0.25),  [0.34, 0.39]  0.37 (0.27),  [0.35, 0.40] | 0.69 (0.43),  [0.63, 0.75]  0.65 (0.34),  [0.61, 0.68]  0.62 (0.34),  [0.60, 0.65]  0.55 (0.31),  [0.52, 0.59] | 7.17 (0.86),  [6.99, 7.35]  6.95 (0.98),  [6.86, 7.05]  6.88 (0.93),  [6.79, 6.96]  6.71 (0.97),  [6.60, 6.82] |

Table 6. 1 (continued) Descriptive statistics of contextual factors (‘The Player’ and ‘The Environment’) and outcome variables in the multivariate analysis (Mean, Standard Deviation, 95% Upper, Lower CI).

|  |  |  | | | | | | | | | |
| --- | --- | --- | --- | --- | --- | --- | --- | --- | --- | --- | --- |
|  |  | **Distance** | | | | | **Acceleration** | | | | **Speed** |
|  | *M, (SE), [95% CI L, U]* | **Total Distance**  **(m)** | **Low Speed Distance**  **(m)** | **Moderate Speed Distance**  **(m)** | **High Speed Distance**  **(m)** | **Very High-Speed Distance**  **(m)** | **Moderate Intensity Acceleration Efforts**  **(#)** | **High Intensity Acceleration Efforts**  **(#)** | **Moderate Intensity Deceleration Efforts**  **(#)** | **High Intensity Deceleration Efforts**  **(#)** | **Maximal Velocity**  (m·s) |
| **The Player** | **Playing Status**  Starter  Sub | 94.42 (12.89), [93.65, 95.18]  94.73 (13.90), [93.16, 96.30] | 59.67 (7.51), [59.21, 60.14]  58.43 (8.44), [57.50, 59.36] | 29.52 (10.17), [28.87, 30.18]  31.14 (12.44), [29.84, 32.44] | 4.43 (4.90), [4.15, 4.72]  4.56 (4.92), [3.98, 5.15] | 0.87 (1.96), [0.75, 0.99]  0.59 (1.79), [0.35, 0.83] | 0.43 (0.26), [0.414, 0.45]  0.46 (0.36), [0.424, 0.49] | 0.25 (0.23), [0.24, 0.27]  0.22 (0.32), [0.19, 0.25] | 0.367 (0.25), [0.35, 0.38]  0.406 (0.38), [0.38, 0.44] | 0.62 (0.32), [0.60, 0.64]  0.62 (0.43), [0.58, 0.66] | 6.96 (0.94),  [6.90, 7.02]  6.62 (0.96),  [6.50, 6.74] |
|  | **Player Position AU I.D.**  Speed Edge  Back    Forward | 93.99 (13.21), [92.57, 95.40]  94.75 (13.08), [93.58, 95.92]  94.53 (12.86), [93.46, 95.60] | 60.95 (7.75), [60.12, 61.79]  58.20 (7.87), [57.51, 58.89]  59.59 (7.06), [58.96, 60.23] | 25.40 (11.24), [24.24, 26.55]  31.10 (10.13), [30.15, 32.06]  31.28 (9.00), [30.41, 32.16] | 5.83 (4.30), [5.32, 6.34]  4.76 (4.58), [4.33, 5.18]  3.41 (5.99), [3.02, 3.80] | 1.83 (1.01), [1.62, 2.03]  0.73 (1.62), [0.57, 0.90]  0.29 (2.95), [0.14, 0.45] | 0.45 (0.28), [0.42, 0.48]  0.48 (0.28), [0.45, 0.50]  0.39 (0.27), [0.37, 0.41] | 0.27 (0.24), [0.25, 0.30]  0.27 (0.25), [0.25, 0.30]  0.20 (0.24), [0.18, 0.22] | 0.35 (0.31), [0.32, 0.37]  0.39 (0.25), [0.37, 0.41]  0.38 (0.26), [0.36, 0.40] | 0.670 (0.33), [0.64, 0.71]  0.689 (0.36), [0.66, 0.72]  0.528 (0.31), [0.50, 0.55] | 7.32 (0.89),  [7.22, 7.43]  6.99 (0.88),  [6.91, 7.08]  6.54 (0.95),  [6.46, 6.62] |
|  | **Player Level**  International  Domestic | 94.71 (12.16), [93.59, 95.83]  94.33 (13.62), [93.46, 95.21] | 59.85 (7.37), [59.18, 60.52]  59.18 (7.89), [58.65, 59.70] | 28.64 (9.21), [27.69,29.59]  30.58 (11.37), [29.84, 31.33] | 5.16 (5.25), [4.75, 5.58]  4.03 (4.63), [3.70 ,4.35] | 1.15 (2.20), [0.97, 1.32]  0.61 (1.72), [0.47, 0.75] | 0.45 (0.26), [0.43, 0.48]  0.42 (0.29), [0.41, 0.44] | 0.34 (0.26), [0.32, 0.36]  0.18 (0.22), [0.17, 0.20] | 0.36 (0.25), [0.34, 0.39]  0.38 (0.30), [0.36, 0.40] | 0.69 (0.34), [0.66, 0.72]  0.57 (0.34),  [0.55, 0.60] | 7.20 (0.89),  [7.11, 7.29]  6.69 (0.95),  [6.63, 6.76] |
| **The Environment** | **Temperature**  Warm  Moderate  Cold | 92.70 (13.41), [91.23, 94.18]  95.32 (13.44), [94.25, 96.39]  94.57 (12.39), [93.45, 95.69] | 57.93 (7.13), [57.05, 58.81]  60.01 (7.64), [59.37, 60.65]  59.66 (7.98), [58.99, 60.33] | 29.20 (10.38), [27.95, 30.45]  30.16 (11.16), [29.25, 31.08]  29.89 (10.20), [28.93, 30.85] | 4.63 (6.52), [4.08, 5.18]  4.35 (4.04), [3.95, 4.75]  4.47 (4.68), [4.05, 4.89] | 0.972 (2.04), [0.74, 1.20]  0.902 (2.02), [0.73, 1.07]  0.624 (1.75), [0.45, 0.08] | 0.44 (0.26), [0.41, 0.47]  0.44 (0.29), [0.42, 0.47]  0.42 (0.29), [0.40, 0.45] | 0.26 (0.23), [0.23, 0.29]  0.26 (0.26), [0.24, 0.28]  0.22 (0.24), [0.20, 0.25] | 0.376 (0.27), [0.35, 0.40]  0.363 (0.29), [0.34, 0.38]  0.385 (0.27), [0.36, 0.41] | 0.62 (0.33), [0.59, 0.66]  0.64 (0.34), [0.61, 0.66]  0.60 (0.35), [0.57, 0.63] | 6.97 (1.01),  [6.86, 7.09]  6.96 (0.92),  [6.87, 7.04]  6.76 (0.95),  [6.67, 6.85] |
|  | **Game Time**  Evening  Afternoon  Morning | 96.63 (13.04), [95.02, 98.23]  94.35 (13.59), [93.22, 95.89]  93.71 (12.58), [92.70, 94.73] | 59.622 (8.11), [58.66, 60.59]  59.534 (7.64),  [58.85, 60.22]  59.273 (7.58), [58.66, 59.89] | 31.00 (9.58), [29.64, 32.36]  29.89 (11.18), [28.92, 30.86]  29.36 (10.57), [28.50, 30.23] | 5.46 (6.94), [4.87, 6.06]  4.19 (4.60), [3.77, 4.61]  4.27 (4.05), [3.89, 4.65] | 0.63 (1.33), [0.38, 0.88]  0.83 (2.01), [0.65, 1.01]  0.87 (2.06), [0.72, 1.03] | 0.430 (0.29), [0.40, 0.46]  0.424 (0.30), [0.40, 0.45]  0.446 (0.27), [0.42, 0.47] | 0.29 (0.25), [0.26, 0.32]  0.23 (0.27), [0.21, 0.25]  0.24 (0.22), [0.22, 0.26] | 0.38 (0.25), [0.35, 0.41]  0.38 (0.32), [0.36, 0.40]  0.37 (0.25), [0.35, 0.39] | 0.68 (0.37), [0.64, 0.72]  0.59 (0.36), [0.56, 0.62]  0.62 (0.32), [0.59, 0.61] | 6.96 (0.92),  [6.83, 7.08]  6.83 (0.96),  [6.74, 6.92]  6.90 (0.96),  [6.82, 6.98] |
