# Supplementary material for: Contextual factors matter: A two-year exploration into the impact of contextual factors on elite women’s rugby sevens match-play movement demands
Source: PLoS One. 2025 May 7;20(5):e0322407. doi: 10.1371/journal.pone.0322407 (PMC12057925; doi:10.1371/journal.pone.0322407)
Supplement: S2 Table — (DOCX) [file pone.0322407.s002.docx]

Supplements Table 2. The Tournament: Univariate Regression Result (Mean >, Mean % Dif, Standard Deviation, 95% Upper Lower CI, P-Value).

|  | **The Tournament: Univariate Analysis** | | | | | | | | | |
| --- | --- | --- | --- | --- | --- | --- | --- | --- | --- | --- |
|  | **Distance** | | | | | **Acceleration** | | | | **Speed** |
| *%MD, (SE), [95% CI L, U], P* | **Total Distance**  **(m)** | **Low Speed Distance**  **(m)** | **Moderate Speed Distance**  **(m)** | **High Speed Distance**  **(m)** | **Very High-Speed Distance**  **(m)** | **Moderate Intensity Acceleration Efforts**  **(#)** | **High Intensity Acceleration Efforts**  **(#)** | **Moderate Intensity Deceleration Efforts**  **(#)** | **High Intensity Deceleration Efforts**  **(#)** | **Maximal Velocity**  (m·s) |
| **Day Number**  **Day 1** *(D1)*  **Day 2** *(D2)* *(Ref)* | **D1** > D2,  3.03 (2.02),  [1.49, 4.21],  P < 0.001 | D1, **D2**,  0.01 (0.01),  [-0.84, 0.82],  P = 0.985 | **D1** > D2,  7.66 (1.61),  [1.12, 3.44],  P > 0.001 | **D1** > D2,  12.11 (0.38),  [0.02, 1.05],  P = 0.04 | **D1,** D2,  1.11 (0.01),  [-0.21, 0.23],  P =0.932 | D1, **D2**,  2.07 (0.01),  [-0.04, 0.02],  P = 0.506 | **D1,** D2,  9.05 (0.02),  [-0.01, 0.05],  P = 0.115 | D1, **D2**,  1.87 (0.00),  [-0.03, 0.02],  P = 0.649 | **D1,** D2,  2.59 (0.01),  [-0.02, 0.05],  P = 0.396 | **D1,** D2,  1.06 (0.05),  [-0.04, 0.18],  P =0.186 |
| **Halves of Play**  **Half 1** *(H1)*  **Half 2** *(H2) (Ref)* | **H1** > H2,  1.6 (1.07),  [0.24, 2.79],  P = 0.020 | **H1**, H2,  1.1 (0.46),  [-0.09, 1.39],  P = 0.084 | **H1,** H2,  1.77 (0.37),  [-0.46, 1.51],  P = .292 | **H1,** H2,  5.28 (0.17),  [-0.23, 0.70],  P = 0.32 | **H1** > H2,  23.58 (0.14),  [0.01, 0.38],  P = 0.036 | **H1,** H2,  3.45 (0.01),  [-0.01, 0.04],  P = 0.293 | **H1** > H2,  15.64 (0.03),  [0.02, 0.06],  P < 0.001 | **H1,** H2,  2.14 (0.01),  [-0.02, 0.04],  P =0.524 | **H1** > H2,  8.09, (0.04),  [0.02, 0.08],  P = 0.002 | **H1** > H2,  4.04 (0.20),  [0.20, 0.36],  P < 0.001 |
| **Match Type**  **Pool** *(P)*  **Final** *(F) (Ref)* | **P** > F,  2.51 (1.67),  [0.96, 3.77],  P < 0.001 | **P,** F,  0.66 (0.28),  [-0.46, 1.24],  P = 0.368 | **P** > F,  5.89 (1.23),  [0.55, 2.95],  P = 0.004 | **P,** F,  5.78 (0.18),  [-0.27, 0.78],  P = 0.343 | P, **F**,  4.16 (0.02),  [-0.26, 0.19],  P = 0.761 | P, **F,**  2.52 (0.01),  [-0.04, 0.02]  P =0.458 | P, **F**,  0.41 (0.00),  [-0.03, 0.03],  P =0.911 | **P,** F,  1.07, (0.00),  [-0.024, 0.03],  P =0.815 | P, **F**,  2.10 (0.01),  [-0.045, 0.03], P=0.481 | P, **F**,  0.38 (0.02),  [-0.14, 0.09],  P = 0.650 |
| *The bolding is showing the direction of the effect. > or < signs and green shading are also used to show significance. | | | | | | | | | | |
