# Supplementary material for: Contextual factors matter: A two-year exploration into the impact of contextual factors on elite women’s rugby sevens match-play movement demands
Source: PLoS One. 2025 May 7;20(5):e0322407. doi: 10.1371/journal.pone.0322407 (PMC12057925; doi:10.1371/journal.pone.0322407)
Supplement: S3 Table — (DOCX) [file pone.0322407.s003.docx]

Supplements Table 3. The Match: Univariate Regression Results (Mean >, Mean % Dif, Standard Deviation, 95% Upper Lower CI, P-Value).

|  | **The Match: Univariate Analysis** | | | | | | | | | |
| --- | --- | --- | --- | --- | --- | --- | --- | --- | --- | --- |
|  | **Distance** | | | | | **Acceleration** | | | | **Speed** |
| *%MD, (SE), [95% CI L, U], P* | **Total Distance**  **(m)** | **Low Speed Distance**  **(m)** | **Moderate Speed Distance**  **(m)** | **High Speed Distance**  **(m)** | **Very High-Speed Distance**  **(m)** | **Moderate Intensity Acceleration Efforts (#)** | **High Intensity Acceleration Efforts**  **(#)** | **Moderate Intensity Deceleration Efforts (#)** | **High Intensity Deceleration Efforts**  **(#)** | **Maximal Velocity**  (m·s) |
| **Result:**  **Draw** *(D)*  **Loss** *(L)*  **Win** *(W) (Ref)* | **W** > L,  2.34 (1.57),  [-3.61, -0.82], P=0.002 | **W** > L,  1.83 (0.77),  [-1.93, -0.26],  P = 0.010 | **W,** L,  1.18 (0.25),  [-1.53, 0.83],  P = 0.558 | **W** > L:  14.88 (0.47),  [-1.18, -0.15),  P = 0.012 | **W,** L,  18.93 (0.11),  [-0.37, 0.07],  P = 0.171 | **W,** L,  4.81 (0.01),  [-0.05, 0.01],  P = 0.162 | **W** > L,  24.95 (0.04)  [-0.09, -0.04], P<0.001 | **W,** L,  1.87 (0.00),  [-0.03, .02],  P = 0.609 | **W** > L,  7.28 (0.03),  [-0.08, -0.01],  P = 0.011 | **W** > L,  2.69 (0.13),  [-0.29, -0.08],  P < 0.001 |
|  | **W,** D,  0.31 (0.21),  [-3.99, 3.40], P=0.876 | **W,** D,  2.87 (1.20),  [-3.92, 0.53], P=0.134 | W, **D**,  1.72 (0.37),  [-2.66, 3.70], P=0.747 | W, **D**,  6.77 (0.24),  [-1.04, 1.72], P=0.632 | W, **D**,  34.23 (0.26),  [-0.22, -0.95], P=0.222 | **W,** D,  4.58 (0.01),  [-0.99, 0.58], P=0.612 | W, **D**,  10.67 (0.02),  [-0.04, -0.10], P=0.397 | **W,** D,  0.53 (0.00),  [-0.07, 0.07], P=0.958 | W, **D**,  5.76 (0.03),  [-0.06, -0.13], P=0.441 | W, **D**,  1.52 (0.08),  [-0.19, 0.40], P=0.482 |
| **Score-line: Margin of Result**  **<14 Points** *(-14)*  **>14 Points** *(+14) (Ref)* | **-14,** +14,  1.04 (0.70),  [-0.38, 2.35],  P =0.158 | **-14,** +14,  0.8 (0.34), [-0.35, 1.29],  P =0.256 | -14, +**14**,  2.09 (0.44), [-1.78, 0.53],  P =0.29 | **-14 >** +14,  17.15 (0.54),  [0.26, 1.27],  P = 0.003 | **-14 >** +14,  41.62 (0.24),  [0.13, 0.55],  P = 0.002 | **-14,** +14,  1.84 (0.01) [-0.02, 0.04],  P = 0.581 | **-14** > +14, 18.03(0.03),  [0.02, 0.07], P < 0.001 | -14, **+14,**  3.47 (0.01), [-0.04, 0.01],  P =0.346 | **-14** > +14,  10.19 (0.04),   [0.03, 0.098],  P < 0.001 | **-14** > +14,  3.88 (0.19), [0.16 0.37],  P < 0.001 |
| **Own Team Ranking**  **Top 5** *(T5)*  **Bottom 5** *(B5) (Ref)* | **T5** > B5,  2.68 (1.8),  [1.08, 4.01],  P < 0.001 | **T5,** B5,  0.48 (0.20) [-0.60, 1.18],  P =0.528 | **T5,** B5,  2.07 (0.44)  [0-.65, 1.89],  P = 0.335 | **T5** > B5,  21.6 (0.71)  [0.45, 1.55],  P < 0.001 | **T5** > B5,  63.39 (0.41) [0.35, 0.81],  P < 0.001 | **T5** > B5,  19.31, (0.06) [0.06, 0.12],  P < 0.001 | **T5** > B5,  44.99 (0.08) [0.09, 0.15],  P < 0.001 | **T5,** B5,  4.24 (0.01) [-0.01, 0.04],  P = 0.277 | **T5** > B5,  21.46 (0.10) [0.10, 0.17],  P < 0.001 | **T5** > B5,  9.09 (0.45) [0.53, 0.75],  P < 0.001 |
| **Opponent Ranking Difference**  **Opponent >4** *(1)*  **Opponent** >1-3 *(2)*  **Opponent** <1-3 *(3)*  **Opponent <4** *(4) (Ref)* | **1** > 4,  3.27 (2.16), [0.31, 5.80],  P = 0.029 | **1,** 4,  0.24 (0.10),  [-1.51, 1.797],  P = 865 | **1,** 4,  5.69 (1.18),  [-0.66, 4.00],  P = 0.160 | **1,** 4:  22.46 (0.68), [-0.06, 1.99],  P = 0.064 | **1,** 4,  33.49 (0.20),  [-0.15, 0.71],  P = 0.203 | **1** > 4,  15.21 (0.05), [0.01, 0.13],  P = 0.02 | **1** > 4,  48.84 (0.09), [0.08, 0.18],  P < 0.001 | **1,** 4,  8.25 (0.02), [-0.02, 0.09],  P = 0.243 | 1 > 4,  21.15 (0.09), [0.06, 0.20],  P < 0.001 | **1** > 4**,**  6.67 (0.33), [0.25, 0.68],  P < 0.001 |
|  | **2** > 4,  3.40 (2.25), [1.31, 5.05],  P < 0.001 | **2** > 4,  1.95 (0.82),  [0.03, 2.29],  P = 0.044 | **2,** 4,  3.55 (0.73),  [-0.56, 2.62],  P = 0.203 | **2** > 4:  18.67 (0.56),  [0.09, 1.49],  P = 0.027 | **2,** 4,  29.7 (0.17), [-0.05, 0.53],  P = 0.105 | **2** > 4,  9.46 (0.03), [0.001, 0.08],  P = 0.04 | **2** > 4,  29.7 (0.05), [0.03, 0.10],  P < 0.001 | **2,** 4,  2.13 (0.01),  [-0.03, 0.04],  P = 676 | **2** > 4,  15.33 (0.07),  [0.04, 0.14],  P < 0.001 | **2** > 4,  3.6 (0.17), [0.10, 0.39],  P < 0.001 |
|  | **3** > 4,  3.73 (2.47),  [1.72, 5.27],  P < 0.001 | **3,** 4,  0.65 (0.27),  [-0.68, 1.45],  P = 0.48 | **3** > 4,  7.76 (1.63),  [0.799, 3.81],  P = 0.003 | **3** > 4,  19.74 (0.59),  [0.17, 1.499],  P = 0.013 | **3,** 4,  9.38 (0.05),  [-0.21, 0.35],  P = 0.629 | 3, **4**,  2.63 (0.01),  [-0.03, 0.05],  P = 0.577 | **3** > 4,  21.1 (0.03), [0.01, 0.08],  P = 0.006 | 3, **4**,  1.9 (0.00),  [-0.04, 0.03],  P = 678 | **3** > 4,  11.56 (0.05),  [0.02, 0.11],  P = 0.003 | **3** > 4,  2.46 (0.12), [0.03, 0.30],  P = 0.017 |
| *The bolding is showing the direction of the effect. > or < signs and green shading are also used to show significance. | | | | | | | | | | |
