# Supplementary material for: Contextual factors matter: A two-year exploration into the impact of contextual factors on elite women’s rugby sevens match-play movement demands
Source: PLoS One. 2025 May 7;20(5):e0322407. doi: 10.1371/journal.pone.0322407 (PMC12057925; doi:10.1371/journal.pone.0322407)
Supplement: S4 Table — (DOCX) [file pone.0322407.s004.docx]

Supplements Table 4 The Player: Univariate Regression Results (Mean >, Mean % Dif, Standard Deviation, 95% Upper Lower CI, P-Value).

|  | **The Player: Univariate Analysis** | | | | | | | | | |
| --- | --- | --- | --- | --- | --- | --- | --- | --- | --- | --- |
|  | **Distance** | | | | | **Acceleration** | | | | **Speed** |
| *%MD, (SE), [95% CI L, U], P* | **Total Distance**  **(m)** | **Low Speed Distance**  **(m)** | **Moderate Speed Distance**  **(m)** | **High Speed Distance**  **(m)** | **Very High-Speed Distance**  **(m)** | **Moderate Intensity Acceleration Efforts**  **(#)** | **High Intensity Acceleration Efforts**  **(#)** | **Moderate Intensity Deceleration Efforts**  **(#)** | **High Intensity Deceleration Efforts**  **(#)** | **Maximal Velocity**  (m·s) |
| **Playing Status**  **Starter** *(ST)*  **Sub** *(SB) (Ref)* | ST, **SB,**  0.33(0.22), [-2.06, 1.43],  P =0.723 | **ST** > SB, 2  .11 (0.88), [0.21, 2.29],  P = 0.019 | **SB** > ST,  5.32 (1.14), [-3.07, -.17],  P = 0.029 | ST, **SB,**  2.93 (0.09), [-0.78, 0.52],  P = 0.69 | **ST** > SB**,**  39.01 (0.20),  [0.02, 0.55],  P = 0.037 | ST, **SB,**  6.09 (0.02), [- .06, 0.01],  P = 0.153 | **ST** = SB,  11.86 (0.02), [-0.004, 0.06],  P = 0.091 | **SB** > ST,  10.09 (0.03), [-0.07, -0.005]  P = 0.025 | ST, **SB**,  0.81 (0.02), [-0.05, 0.04],  P = 0.829 | **ST** > SB,  4.98 (0.24), [0.21, 0.47],  P < 0.001 |
| **Player Position AU I.D.**  **Speed Edge** *(SE)*  **Back** *(B)* **Forward** *(F) (Ref)* | **F,** SE,  0.58 (0.39),  [-2.32, 1.23],  P = .546 | **F** > SE,  2.25 (0.96), [0.31, 2.403],  P = 0.01 | **F** > SE,  20.77 (4.16),  [-7.33, -4.44],  P < 0.001 | **SE** > F,  52.48 (1.71), [1.79, 3.06],  P < 0.001 | **SE** > F,  144.85 (1.08), [1.28, 1.79],  P < 0.001 | **SE** > F,  13.37 (0.04) [0.019, 0.09],  P = 0.03 | **SE** > F,  29.05 (0.05),  [0.04, 0.103],  P < 0.001 | **F,** SE,  8.02 (0.02), [-0.06, 0.01],  P = 0.09 | **SE** > F,  27.31 (0.10), [0.099, 0.19],  P < 0.001 | **SE** > F,  11.31 (0.55), [0.65, 0.91],  P < 0.001 |
|  | F, **B**,  0.23 (0.15), [-1.37, 1.81],  P = .788 | **F** > B,  2.37 (0.99), [-2.33, -0.46],  P = 0.04 | F, **B,**  0.57 (0.13),  [-1.48, 1.12],  P = 0.786 | **B** > F,  32.96 (0.95), [0.77, 1.92],  P < 0.001 | **B** > F,  86.05 (0.31), [0.21, 0.67],  P < 0.001 | **B** > F,  20.02 (0.06), [0.05, 0.12],  P < 0.001 | **B** > F,  29.77 (0.05),  [0.04, 0.101],  P < 0.001 | F, **B**,  3.91 (0.01),  [-0.02, 0.04],  P = 0.356 | **B** > F,  26.46 (0.11), [0.12, 0.199],  P < 0.001 | **B** > F,  6.74 (0.32),  [0.34, 0.57],  P < 0.001 |
| **Player Level**  **Int** *(I)*  **Dom** *(D) (Ref)* | **I,** D,  0.4 (0.27),  [-1.04, 1.79],  P = 0.6 | **I,** D**,**  1.13 (0.48), [-0.18, 1.53],  P = 0.12 | **D** > I,  6.58 (1.38), [-3.15, -0.74],  P = 0.02 | **I** > D,  24.71 (0.80), [0.61, 1.67],  P < 0.001 | **I** > D,  61.12 (0.38), [0.32, 0.76],  P < 0.001 | **I,** D,  6.83 (0.02), [-0.00, 0.06],  P = 0.052 | **I** > D,  59.94 (0.11), [0.13, 0.19],  P < 0.001 | I, **D,**  4.84 (0.01), [-0.05, 0.01],  P = 0.21 | **I** > D,  18.64 (0.08), [0.08, 0.16],  P < 0.001 | **I** > D,  7.28 (0.36), [0.397, 0.62],  P < 0.001 |
| *The bolding is showing the direction of the effect. > or < signs and green shading are also used to show significance. | | | | | | | | | | |
