# Supplementary material for: Contextual factors matter: A two-year exploration into the impact of contextual factors on elite women’s rugby sevens match-play movement demands
Source: PLoS One. 2025 May 7;20(5):e0322407. doi: 10.1371/journal.pone.0322407 (PMC12057925; doi:10.1371/journal.pone.0322407)
Supplement: S5 Table — (DOCX) [file pone.0322407.s005.docx]

Supplements Table 5 The Environment: Univariate Regression Result (Mean >, Mean % Dif, Standard Deviation, 95% Upper Lower CI, P-Value)

|  | **The Environment: Univariate Analysis** | | | | | | | | | |
| --- | --- | --- | --- | --- | --- | --- | --- | --- | --- | --- |
|  | **Distance** | | | | | **Acceleration** | | | | **Speed** |
| *‘%MD, (SE), [95% CI L, U], P* | **Total Distance**  **(m)** | **Low Speed Distance**  **(m)** | **Moderate Speed Distance**  **(m)** | **High Speed Distance**  **(m)** | **Very High-Speed Distance**  **(m)** | **Moderate Intensity Acceleration Efforts**  **(#)** | **High Intensity Acceleration Efforts**  **(#)** | **Moderate Intensity Deceleration Efforts**  **(#)** | **High Intensity Deceleration Efforts**  **(#)** | **Maximal Velocity**  (m·s) |
| **Temperature**  **Warm** *(W)*  **Moderate** *(M)*  **Cold** *(C) (Ref)* | **C** > W**,**  1.99 (1.32), [-3.72, -0.02],  P = 0.048 | **C** > W,  2.94 (1.22), [-2.83, -0.62],  P = 0.002 | **C,** W,  2.35 (0.49), [-2.27, 0.88],  P = 0.388 | C, **W,**  3.49 (0.11), [-0.53, 0.85],  P = 0.652 | **W** > C,  43.61 (0.25), [0 .06, 0.64],  P = 0.018 | C, **W**,  3.25 (0.01), [-0.03, 0.05],  P = 0.47 | C, **W**,  14.17 (0 .02), [-0.00, 0.07],  P = 0.058 | **C,** W,  2.37 (0.01),  [-0.05, 0.03],  P = 0.586 | C, **W**,  4.26 (0.02),  [-0.003, 0.08],  P = 0.277 | **W** > C,  3.10 (0.15), [0.08, 0.32],  P = 0.004 |
|  | C, **M**,  0.79 (0.53),  [-0.80, 2.298],  P = 0.344 | C, **M**,  0.59 (0.25),  [-0.57, 1.28],  P = 0.454 | C, **M**,  0.91 (0.19),  [-1.05, 1.597],  P = 0.688 | **C,** M,  2.74 (0.09),  [-0.70, 0.46],  P = 0.682 | **M** > C,  36.44 (0.20), [0.04, 0.52],  P = 0.025 | C, **M**,  4.61 (0.01),  [-0.01, 0.05],  P = 0.245 | **M** > C,  14.17 (0.02), [0.004, 0.06],  P = 0.025 | **C,** M**,**  5.88 (0.02), [-0.05, 0.01],  P = 0.149 | C, **M**,  6.00 (0.03) [-0.003, 0.08],  P = 0.07 | **M** > C,  2.89 (0.14) [0.07, 0.36],  P = 0.002 |
| **Game Time**  **Evening** *(E)*  **Afternoon** *(A)*  **Morning** *(M) (Ref)* | **E** > M,  3.07 (2.06), [1.02, 4.81],  P = 0.003 | **E,** M,  0.59 (0.25), [-0.79, 1.49],  P = 0.549 | **E** > M,  5.43 (1.16),  [0.03, 3.25],  P = 0.046 | **E** > M,  24.5 (0.84), [0.49, 1.897],  P < 0.001 | **M,** E,  32.91 (0.17), [-0.54, 0.05],  P = 0.101 | **M,** E,  3.65 (0.01), [-0.06, 0.03],  P = .457 | **E** > M,  21.21 (0.04), [0.02, 0.092],  P = 0.002 | **E,** M,  4.01 (0.01), [-0.02, 0.05],  P = 0.436 | **E** > M,  9.58 (0.04), [0.01, 0.11],  P = 0.012 | **E,** M,  0.76 (0.04), [-0.096, 0.202],  P = 0.487 |
|  | M, **A**,  0.68 (0.45),  [-0.87, 2.16],  P = 0.407 | M, **A**,  0.44 (0.18),  [-0.65, 1.17],  P = 0.575 | M, **A**,  1.79 (0.38),  [-0.76, 1.82],  P = 0.420 | **M,** A,  1.89 (0.06),  [-0.64, 0.48],  P = 0.781 | **M,** A,  5.41 (0.03),  [-0.28, 0.29],  P = 0.702 | **M,** A,  5.06 (0.02),  [-0.05, 0.01],  P = 0.201 | **M,** A,  2.58 (0.00),  [-0.03, 0.02],  P = 0.725 | M, **A**,  3.22 (0.01),  [-0.02, 0.04],  P = 0.448 | **M,** A,  3.80 (0.02),  [-0.06, 0.02],  P = 0.258 | **M,** A,  1.05 (0.05),  [-0.19, 0.05],  P = 0.233 |
| *The bolding is showing the direction of the effect. > or < signs and green shading are also used to show significance. | | | | | | | | | | |
