# Supplementary material for: Contextual factors matter: A two-year exploration into the impact of contextual factors on elite women’s rugby sevens match-play movement demands
Source: PLoS One. 2025 May 7;20(5):e0322407. doi: 10.1371/journal.pone.0322407 (PMC12057925; doi:10.1371/journal.pone.0322407)
Supplement: S6 Table — (DOCX) [file pone.0322407.s006.docx]

## Table 9.5 Multivariate Reduction Process of contextual factors (Model number, contextual factor, Akaike Information Criterion (AIC), Bayesian Information Criterion (BIC)).

| **Total Distance** | | | | | |
| --- | --- | --- | --- | --- | --- |
| **Model #** | **Contextual Factor** | **Reason for removal**  **(P > 0.05)** | **AIC** | **BIC** | **Variable to be removed** |
| #1 - Full | Day Number Half Number Match Type Result WLD Ranking - Own Team Ranking Difference Temperature Match Time | P = 0.12 & 0.637 / Highest P-Values from List | 11474.99 | 11485.537 | Temperature |
| #2 | Day Number Half Number Match Type Result WLD Ranking - Own Team Ranking Difference Match Time | P = 0.66 & 0.789 / Highest P-Values from List | 11481.81 | 11492.355 | Match Time |
| #3 | Day Number Half Number Match Type Result WLD Ranking - Own Team Ranking Difference | P = 0.860 / Highest P-Values from List | 11485.12 | 11495.673 | Match Type |
| #4 | Day Number Half Number Result WLD Ranking - Own Team Ranking Difference | P = 0.051 / Highest P-Values from List | 11488.27 | 11498.822 | Half Number |
| #5 - Final | Day Number Result WLD Ranking - Own Team Ranking Difference | All P's < 0.05 | 11548.9 | 11559.4665 | **Final** |
| **Low Speed Distance - Zone 1** | | | | | |
| **Model #** | **Contextual Factor** | **Reason for removal**  **(P > 0.05)** | **AIC** | **BIC** | **Variable to be removed** |
| #1 - Full | Half Number Result WLD Ranking Difference Playing Status Player Position Temperature | P = 0.679 & 0.686 & 0.555 / Highest P-Values from List | 9957.921 | 9968.468 | Ranking Difference |
| #2 | Half Number Result WLD Ranking Difference Playing Status Player Position Temperature | P = 0.462 / Highest P-Values from List | 9952.087 | 9962.638 | Half Number |
| #3 - Final | Result WLD Ranking Difference Playing Status Player Position Temperature | All P's < 0.05 | 10008.08 | 10018.638 | **Final** |
| **Moderate Speed Distance - Zone 2** | | | | | |
| **Model #** | **Contextual Factor** | **Reason for removal**  **( P >0.05)** | **AIC** | **BIC** | **Variable to be removed** |
| #1 - Full | Day Number Match Type Ranking Difference Playing Status Player Position Player Level Match Time | P = 0.999 & 0.944 / Highest P-Values from List | 10889.95 | 10900.503 | Match Time |
| #2 | Day Number Match Type Ranking Difference Playing Status Player Position Player Level | P = 0.661 / Highest P-Values from List | 10892.25 | 10902.812 | Match Type |
| #3 | Day Number Ranking Difference Playing Status Player Position Player Level | P = 0.087 / Highest P-Values from List | 10894.44 | 10905.004 | Playing Status |
| #4 - Final | Day Number Ranking Difference Player Position Player Level | All P's < 0.05 | 10898.55 | 10909.1077 | **Final** |
| **High Speed Distance - Zone 3** | | | | | |
| **Model #** | **Contextual Factor** | **Reason for removal**  **(P > 0.05)** | **AIC** | **BIC** | **Variable to be removed** |
| #1 - Full | Day Number Result WLD Result Margin Ranking - Own Team Ranking Difference Player Position Player Level Match Time | P = 0.234, 0.771 & 0.882 / Highest P-Values from List | 8624.617 | 8635.174 | Ranking Difference |
| #2 | Day Number Result WLD Result Margin Ranking - Own Team Player Position Player Level Match Time | P = 0.994 / Highest P-Values from List | 8623.854 | 8634.412 | Match Time |
| #3 | Day Number Result WLD Result Margin Ranking - Own Team Player Position Player Level | P = 0.194 / Highest P-Values from List | 8630.252 | 8640.813 | Ranking - Own Team |
| #4 | Day Number Result WLD Result Margin Player Position Player Level | P = 0.883 / Highest P-Values from List | 8631.403 | 8641.966 | Result WLD |
| #5 | Day Number Result Margin Player Position Player Level | P = 0.055 / Highest P-Values from List | 8636.28 | 8646.846 | Result Margin |
| #6 - Final | Day Number Result Margin Player Position Player Level | All P's < 0.05 | 8643.107 | 8653.673 | **Final** |
| **Very High-Speed Distance - Zone 4** | | | | | |
| **Model #** | **Contextual Factor** | **Reason for removal**  **(P > 0.05)** | **AIC** | **BIC** | **Variable to be removed** |
| #1 - Full | Half Number Result Margin Ranking - Own Team Playing Status Player Position Player Level Temperature | P = 0.963 / Highest P-Values from List | 5895.846 | 5906.396 | Playing Status |
| #2 | Half Number Result Margin Ranking - Own Team Player Position Player Level Temperature | P = 0.196 / Highest P-Values from List | 5893.717 | 5904.269 | Half Number |
| #3 | Result Margin Ranking - Own Team Player Position Player Level Temperature | P = 0.68 / Highest P-Values from List | 5914.697 | 5925.26 | Player Level |
| #4 - Final | Result Margin Ranking - Own Team Player Position Temperature | All P's < 0.05 | 5915.462 | 5926.027 | **Final** |
| **Acceleration Efforts - Moderate Z2** | | | | | |
| **Model #** | **Contextual Factor** | **Reason for removal**  **(P > 0.05)** | **AIC** | **BIC** | **Variable to be removed** |
| #1 - Full | Ranking - Own Team Ranking Difference Player Position Player Level | P = 0.478, 0.834, 0.721 / Highest P-Values from List | 339.286 | 349.849 | Ranking Difference |
| #2 | Ranking - Own Team Player Position Player Level | P = 0.694 / Highest P-Values from List | 322.311 | 332.878 | Player Level |
| #3 - Final | Ranking - Own Team Player Position | All P's < 0.05 | 316.05 | 326.619 | **Final** |
| **Acceleration Efforts - High - Z3** | | | | | |
| **Model #** | **Contextual Factor** | **Reason for removal**  **(P > 0.05)** | **AIC** | **BIC** | **Variable to be removed** |
| #1 - Full | Half Number Result WLD Result Margin Ranking - Own Team Ranking Difference Playing Status Player Position Player Level Temperature Match Time | P = 0.527, 0.545, 0.691 / Highest P-Values from List | -339.758 | -329.217 | Ranking Difference |
| #2 | Half Number Result WLD Result Margin Ranking - Own Team Playing Status Player Position Player Level Temperature Match Time | P = 0.225, 0.473 / Highest P-Values from List | -355.854 | -345.309 | Result WLD |
| #3 | Half Number Result Margin Ranking - Own Team Playing Status Player Position Player Level Temperature Match Time | P = 0.074 / Highest P-Values from List | -365.191 | -354.643 | Playing Status |
| #4 - Final | Half Number Result Margin Ranking - Own Team Player Position Player Level Temperature Match Time | All P's < 0.05 | -368.391 | -357.842 | **Final** |
| **Deceleration Efforts - Moderate Z2** | | | | | |
| **Model #** | **Contextual Factor** | **Reason for removal**  **(P > 0.05)** | **AIC** | **BIC** | **Variable to be removed** |
| #1 - Full | Playing Status Player Position | P = 0.102, 0.302 / Highest P-Values from List | 194.269 | 204.837 | Player Position |
| #2 - Final | Playing Status | All P's < 0.05 | 187.429 | 198 | **Final** |
| **Deceleration Efforts - High - Z3** | | | | | |
| **Model #** | **Contextual Factor** | **Reason for removal**  **(P > 0.05)** | **AIC** | **BIC** | **Variable to be removed** |
| #1 - Full | Half Number Result WLD Result Margin Ranking - Own Team Ranking Difference Player Position Player Level Match Time | P = 0.268, 0.689 , 0.629 / Highest P-Values from List | 817.97 | 828.515 | Ranking Difference |
| #2 | Half Number Result WLD Result Margin Ranking - Own Team Player Position Player Level Match Time | P = 0.559 , 0.505 / Highest P-Values from List | 804.232 | 814.78 | Result WLD |
| #3 - Final | Half Number Result Margin Ranking - Own Team Player Position Player Level Match Time | All P's < 0.05 | 794.339 | 804.891 | **Final** |
| **Maximum Velocity** | | | | | |
| **Model #** | **Contextual Factor** | **Reason for removal**  **(P > 0.05)** | **AIC** | **BIC** | **Variable to be removed** |
| #1 - Full | Half Number Result Margin Ranking - Own Team Ranking Difference Playing Status Player Position Player Level Match Time | P = 0.798 , 0.606 / Highest P-Values from List | 3650.028 | 3660.574 | Match Time |
| #2 | Half Number Result Margin Ranking - Own Team Ranking Difference Playing Status Player Position Player Level | P = 0.616 / Highest P-Values from List | 3642.76 | 3653.309 | Playing Status |
| #3 | Half Number Result Margin Ranking - Own Team Ranking Difference Player Position Player Level | P = 0.084 / Highest P-Values from List | 3639.361 | 3649.912 | Ranking Difference |
| #4 - Final | Half Number Result Margin Ranking - Own Team Player Position Player Level | All P's < 0.05 | 3634.303 | 3644.857 | **Final** |
